# Optimization of the angle for scattered light measurements in 3D-printed cultivation vessels

**DOI:** 10.1007/s00216-025-06131-4

**Published:** 2025-10-02

**Authors:** Nicolas Debener, Louis Maximilian Kuhnke, Sascha Beutel, Janina Bahnemann

**Affiliations:** 1 Institute of Technical Chemistry, Leibniz University Hannover, Hannover, Germany; 2 Institute of Physics, University of Augsburg, Augsburg, Germany; 3 Centre for Advanced Analytics and Predictive Sciences (CAAPS), University of Augsburg, Augsburg, Germany

**Keywords:** 3D printing, Optical waveguide, Light scattering, Online monitoring

## Abstract

Monitoring key parameters during the cultivation of microorganisms—including biomass concentration—is often a crucial prerequisite for attaining reproducible results in the field of biotechnology. In order to overcome the drawbacks associated with conventional methods for assessing biomass concentration (such as cell dry weight or optical density measurements), several devices to facilitate continuous online monitoring have been developed. Yet despite the success of innovative solutions (such as acceleration sensors) in addressing the challenging dynamical behavior of liquids within shaken systems, the performance of these sensors can still be affected by reflections at the liquid–air interface or at the top of the cultivation vessels. In our previous work, a 3D-printed cultivation vessel equipped with modified optical waveguide paths was utilized in an effort to overcome this challenge, allowing for the measurement of scattered light in a lateral direction. In this work, we developed an optical adapter that allows for the rapid assessment of the optimal measurement angle of excitation and detection within these 3D-printed cultivation vessels. The findings of the present study indicate that an angle of 110° yields higher signal intensities and enhanced sensitivity in comparison to larger angles, and this finding was additionally confirmed for a set of biotechnologically relevant microorganisms. While these results suggest that the angle of 110° should be integrated into the cultivation vessels in the future, the optical adapter also holds the potential to further investigate vessels with different sizes or geometries.

**Graphical abstract:**

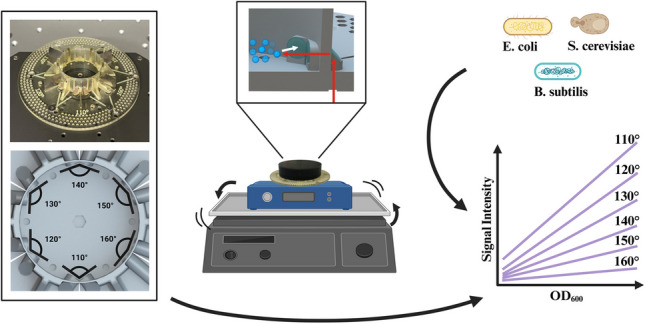

**Supplementary Information:**

The online version contains supplementary material available at 10.1007/s00216-025-06131-4.

## Introduction

Shake flasks play an important role within the field of biotechnology—indeed, they are often used for the cultivation of bacteria, fungi, and yeast, as well as both animal and plant cells [1]. The simplicity of shake flasks enables the simultaneous execution of many different types of experiments. Areas of application include, without limitation, strain development, the screening of wild-type strains, and media optimization [2]. In order to optimize culture conditions and to allow for reproducible experiments, however, the ability to monitor several different culture parameters—including dissolved oxygen (DO), carbon dioxide, and biomass—is highly advantageous [3]. Through biomass measurements, information on the cell growth inside the shake flasks can be obtained which is beneficial for inducing protein expression [4].

Standard approaches, such as dry cell weight measurements or plating for colony-forming units, can be used to obtain information on microbial growth within cultivations. Another common technique is to determine the optical density (OD) by measuring the transmittance of a culture sample to approximate biomass concentration [5–7]. Unfortunately, these methods necessitate sampling from the flasks, which is a relatively time-consuming process that can hinder the feasibility of parallelization and pose significant contamination risks [8]. Furthermore, the process of sample preparation entails a series of manual labor steps, such as sample washing or the preparation of dilutions, which increase the risk of introducing errors [4, 7, 9]. To address these limitations, several different techniques for online biomass measurements have been proposed, including both infrared spectroscopy [10, 11] and fluorescence-based methods [12]. Another frequently used method is the non-invasive measurement of scattered light, which has been performed for various applications involving the cultivation of microorganisms in shake flasks [13]. In comparison to transmission-based turbidity measurements, which exhibit a limited linear range due to the Lambert–Beer law, this approach benefits from increased linearity in higher optical density regions (OD > 0.5) [14]. While scattered light measurements overcome the drawback of contamination risks and allow for continuous online measurements during the cultivation, the dynamic behavior of liquids inside the system leads to varying local filling volumes due to the rotating fluid sickle when orbitally shaken, and the reflections occurring at the liquid–air interface make this approach regrettably prone to errors [15].

The dynamic behavior of the liquid can be compensated by stopping the shaking of the system in order to perform scattered light measurements, but this also affects the growth characteristics of the microorganisms within the culture [16]. Two commercially available systems for online biomass determination that rely on different strategies are the cell growth quantifier (CGQ) and the shake flask reader (SFR vario) produced by Aquila Biolabs GmbH and PreSens Precision Sensing GmbH, respectively. Rather than interrupting the shaking process to conduct measurements, the CGQ device acquires multiple readings to represent the dynamic fluid distribution across multiple shaking periods [17]. In the PreSens SFR vario, an acceleration sensor is employed to predict the agitation of the liquid inside the shake flasks, thereby enabling triggered measurements at time points with high fluid levels above the optics [9, 18]. In order to further reduce disturbing factors such as reflections at the top of the shake flasks or at the liquid–air interface (a schematic illustration can be found in the supplementary material SI1), a 3D-printed cultivation vessel with modified optical waveguides was developed in our previous work [19]. Instead of measuring from the bottom of the shake flasks (as performed in the SFR vario and CGQ), the light in this device is deflected at a 90° angle by an integrated prism and therefore introduced laterally through the shake flask’s wall. This system has been successfully used to cultivate and monitor the growth of different microorganisms and mammalian cells [20].

The aim of the present work was to further improve the signal quality of the lateral scattered light measurements within 3D-printed cultivation vessels. In order to achieve this, a 3D-printed optical adapter was developed that can be mounted onto the PreSens SFR vario device and which features six measurement angles—ranging from 110 to 160°. By correlating the signal intensities with offline-determined optical density values, the adapter facilitates a rapid assessment of the optimal measurement angle for excitation and detection within these vessels. In this work, the impact of the optical waveguide length and the measurement angle on the signal quality was initially evaluated independently of each other, and then subsequently examined for several biotechnologically relevant microorganisms.

## Methods

### Fabrication of the optical adapters

The 3D-printed parts presented in this work were designed using the software Autodesk Inventor Professional 2024 (Autodesk Inc., SanRafael, USA). The adapters and a lid that prevents spillage were fabricated with the high-resolution multijet 3D printer AGILISTA 3200W (Keyence Deutschland GmbH, Neu-Isenburg, Germany) using AR-M2 and AR-S1 (Keyence Deutschland GmbH) as printing and supporting material, respectively. After the printing process, the parts were separated from the printing platform and post-processed as previously described [21]. Briefly summarized, the components were cleaned in an ultrasonic water bath—initially with, and then subsequently without, the use of detergent. Additionally, all surfaces were polished, first with 70% (v/v) isopropanol and consecutively with distilled water. Using the UV-curing lacquer luxaprint shellac (DETAX, Ettlingen, Germany), glass slides (5 mm diameter) were glued into recesses at the underside of the optical adapters. Additionally, glass slides (4 mm diameter) were affixed to the end of the optical waveguides on the inside of the vessel, as well as to the 45° inclined surfaces of the waveguides outside of the vessel. Stainless-steel sheets were cut into 14 × 14 mm pieces to be placed between the waveguides for excitation and detection of each measurement angle. In this study, three adapters with different base thicknesses were fabricated.

### Optical bench setup

The influence of the waveguide length on the detected signal intensity was assessed using an optical bench that was modified from Kuhnke et al. [19]. In brief, a 3D-printed body was made from polylactic acid (PLA) using the fused deposition modeling (FDM) printer MakerBot Replicator Z18 (MakerBot Industries LLC, New York City, USA). The optical bench consists of a liquid crystal light valve (Adafruit Industries LLC, New York City, USA) that changes the amplitude of a laser (a modulated red dot laser, module MI650-1-5, Picotronic GmbH, Koblenz, Germany). Optical waveguides of varying lengths (30, 35, 40, and 45 mm) were then fabricated using the 3D printer AGILISTA 3200W (Keyence Deutschland GmbH) and post-processed as described above. Glass slides (4 mm diameter) were affixed to both ends of each waveguide using the UV-curing lacquer luxaprint shellac (DETAX). These waveguides were aligned between the laser and a Si PIN photodiode (Hamamatsu Photonics K.K., Hamamatsu, Japan) in order to position them to measure the transmitted light, and a plastic cover was used to exclude ambient light during the measurements. A schematic illustration of the setup is shown in Fig. SI2. For each waveguide, signals were recorded for 2.2 s, resulting in approximately 16,000 data points, using the oscilloscope of the STEMlab Red Pitaya board (Red Pitaya, Solkan, Slovenia) and the signal generator JOY-IT JDS2915 (SIMAC Electronics GmbH, Neukirchen-Vluyn, Germany; Settings: 5.6 V amplitude, 100 Hz, offset = 0, duty = 50%).

### Cultivation

Shake flasks (250 mL with baffles) were filled with 50 mL lysogeny broth (LB) medium and inoculated with *Escherichia coli* BL21 DE3 (Thermo Fisher Scientific Inc., Waltham, USA) and *Bacillus subtilis* DSM 23778 (German Collection of Microorganisms and Cell Cultures GmbH, DSMZ, Braunschweig, Germany) from 20% (v/v) glycerol stocks stored at −80 °C. Cultures were grown overnight at 30 °C at 150 rpm. *Saccharomyces cerevisiae* NCYC 1024 (National Collection of Yeast Cultures, Norwich, UK) was grown in Yeast-Peptone-Dextrose (YPD) media in 250-mL shake flasks with 50 mL filling volume for 24 h at 30 °C at 150 rpm.

### Scattered light measurements in the optical adapters

Stationary cell cultures were centrifuged at 4.000 × g and 4 °C for 15 min, and the resulting cell pellets were washed and resuspended in phosphate-buffered saline (PBS, pH 7.4). Sequential dilutions were prepared with PBS to cover an optical density range at 600 nm (OD_600_) of 0–20 rel. AU and measured in *d* = 1 cm cuvettes (Sarstedt AG & Co.KG, Nümbrecht, Germany) at 600 nm in a photometer (Libra S80, Biochrom GmbH, Cambridge, UK).

In preparation for the scattered light measurements, the optical adapter was mounted onto the SRF vario (Precision Sensing GmbH, Regensburg, Germany) and secured with screws. A volume of 4 mL of the sample solution was transferred into the adapter’s vessel. Stainless-steel shields were positioned between the exciting and detecting waveguides outside of the vessel. To prevent spillage of the contents, the adapter’s top was closed using a 3D-printed lid, and the system was shielded from ambient light by a plastic cover. The SFR vario was mounted onto an orbital shaker, and the entire system was shaken at 80 rpm.

The samples were measured for 90 s per angle (LED peak wavelength of 630 nm), resulting in the acquisition of five data points. After each measurement was taken, the shaker was stopped and the adapter was rotated and fixed at the next position to measure the next angle. In order to ensure adequate mixing of the sample, the plastic cover was removed and the sample was mixed via pipetting. Once the sample was measured for all angles, the liquid was taken out of the adapter and the OD_600_ was measured as described above.

## Results and discussion

### Design of the optical adapter

**Fig. 1:**
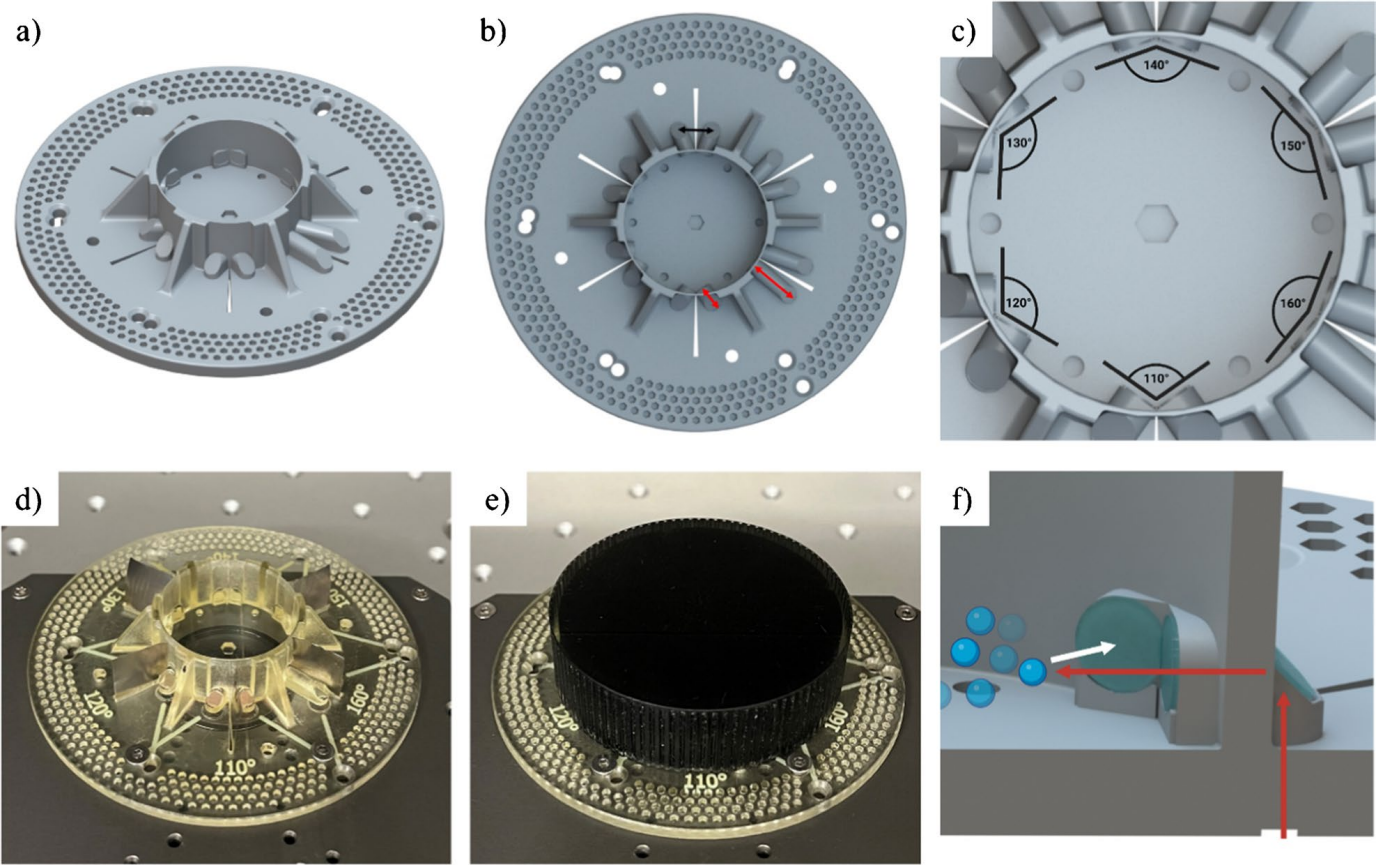
Design of the optical 3D-printed adapter. **a** Schematic overview of the adapter and the reaction vessel. **b** Top view of the optical adapter. The black arrow indicates the distance between the optics, and the red arrows indicate the differences in waveguide length. **c** Magnification of the cultivation vessel with indicated angles of excitation and detection. **d** Photograph of the optical adapter with stainless steel sheets. **e** Photograph of the fully assembled adapter with all additional parts. **f** Cross-sectional view of the cultivation vessel with indicated light path

The design of the optical adapter is shown in Fig. 1. The device consists of a circular base with holes for screws to allow for it to be mounted onto the SFR vario platform. When affixed by screws, the optical waveguides of the adapter were situated in direct alignment to the optics of the SFR vario. A vessel with a volume of approximately 14 mL was located in the center of the device. The adapter features 12 waveguides that allow for the measurement of scattered light at six distinct angles of excitation and detection, ranging from 110 to 160° (Fig. 1a–c). The upper limit for measuring angles is determined by the design of the optical adapter. Measurement angles > 160° necessitate light guides that are longer than can be easily accommodated by the 3D-printed base. For each of the six measurement angles of excitation and detection, two holes are provided in the adapter’s base for the screws. Each waveguide consists of a round base with a slight recess at the underside of the adapter, into which a glass slide is adhered to enhance the transparency at the adapter’s border. Additionally, a glass slide is also affixed to the surface at a 45° angle and to the inner border of the waveguide (Fig. 1f)—which was shown to increase the optical properties of the waveguides [19]. The positioning of stainless-steel pieces into slits between each waveguide pair (excitation and detection) served to prevent the exciting light from being directly measured via the detection channel. In order to avert the potential leakage of liquid from the vessel (particularly during agitation), a 3D-printed lid was added to the device. Furthermore, the use of a plastic cover served to prevent any ambient light from influencing the measurements (Fig. 1e). Due to the possibility to easily adapt the designs of 3D-printed parts, we note that the presented adapter could easily be adjusted to examine cultivation vessels with different sizes or geometries—thus allowing, for example, the examination of the optimal measurement angles for cultivation vessels with custom baffles.

### Influence of the length of the waveguides

In order to measure scattered light with the SFR vario, the excitation and detection optics outside the vessel must be positioned at a specific distance. This distance is consistent across all measurement angles and is dictated by the position of the optics of the SFR vario (as indicated by the black arrow in Fig. 1b). For larger measurement angles inside the vessel, the angle between the corresponding waveguides outside the vessel is smaller. Consequently, the optics must be positioned further away from the vessel in order to achieve the aforementioned distance, resulting in longer waveguides when compared to shorter measurement angles (indicated by red arrows in Fig. 1b).

**Fig. 2:**
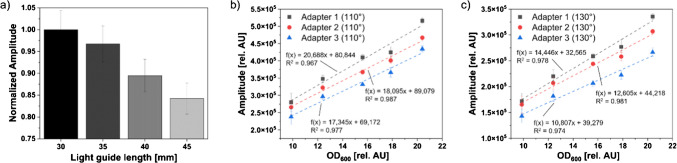
Influence of the waveguide length on the signal intensity. **a** Signal intensities measured through waveguides of varying length with an optical bench. The signals are normalized to the highest intensity, which was obtained for the waveguide with 30 mm length. **b** SFR vario measurements of *E. coli* in the three adapters with different base thicknesses for an angle of 110°. **c** SFR vario measurements of *E. coli* in the three adapters with different base thicknesses for an angle of 130°. Three separate *E. coli* sample solutions were measured for each data point; mean signal intensities and standard deviations are shown in **b** and **c**

To investigate the impact of the waveguide length on the detection signal intensity, straight waveguides of varying length were 3D-printed and the transmission of a laser beam was assessed using an optical bench modified from [19]. The signal intensity was measured by a photodiode placed behind the waveguides, which decreased with increasing waveguide length (Fig. 2a). Waveguides with a length of 45 mm showed 15.8% lower detected signal intensities in comparison to waveguides of 30 mm length. The optical loss determined by these experiments was 0.84 dB/cm, which is lower than the reported losses for waveguides fabricated with FDM printers [22, 23]. By using more complicated fabrication methods, several studies describe waveguides with lower optical losses [24, 25], even reaching values below 0.1 dB/cm [26]. However, given this system’s other requirements—such as the ability to print the entire reaction vessel and light guides using a single material or the thermal stability of the material to enable autoclaving of the system—the optical loss of the waveguides utilized in this study is considered acceptable. A more thorough examination of the waveguides utilized in this study can be found in Kuhnke et al. [19].

In order to assess the influence of the waveguide length on the signal intensity in the final measurement system, the optical adapter’s base was systematically varied. Adapters with three distinct base thicknesses were fabricated, resulting in waveguides of varying lengths, as detailed in Table 1. The model organism *E. coli* was used to prepare three separate sets of sample solutions in the OD_600_ range of 10–20 rel. AU, which were measured with all three adapters for the angles of 110° (Fig. 2b) and 130° (Fig. 2c). For both measurement angles, the mean signal intensities in the medium and thick adapters were observed to be 6.9% (± 2.0%) and 17.4% (± 2.3%) lower, respectively, across the measured OD_600_ in comparison to the thin adapter, while the slopes of the linear fits were found to be 12.6% and 20.7% lower, respectively.

The findings outlined in this section indicate that the length of the waveguides exerts a substantial impact on the signal intensity. In order to achieve higher signal-to-noise ratios (SNR), this factor should be minimized as much as practicable. This objective could be accomplished, for instance, by decreasing the thickness of the adapter’s base and the vessel wall. In previous studies, however, it has been suggested that cultivation vessels made of the 3D printing material used in this work should be autoclaved twice in order to sterilize the system and also enhance its biocompatibility when cultivating Chinese Hamster Ovary (CHO) cells [20]. Due to the deformation of the material at high temperatures, the thickness of supporting structures should be adjusted carefully.

**Table 1:** Overview of the waveguide length of adapters with different base thicknesses

Angle (°)	Length of waveguides (mm), Adapter 1	Length of waveguides (mm), Adapter 2	Length of waveguides (mm), Adapter 3
110	9.94	10.70	11.68
120	10.09	10.85	11.83
130	10.70	11.46	12.44
140	11.68	12.44	13.42
150	13.43	14.19	15.18
160	17.22	17.98	18.96

### Influence of the measurement angle

**Fig. 3:**
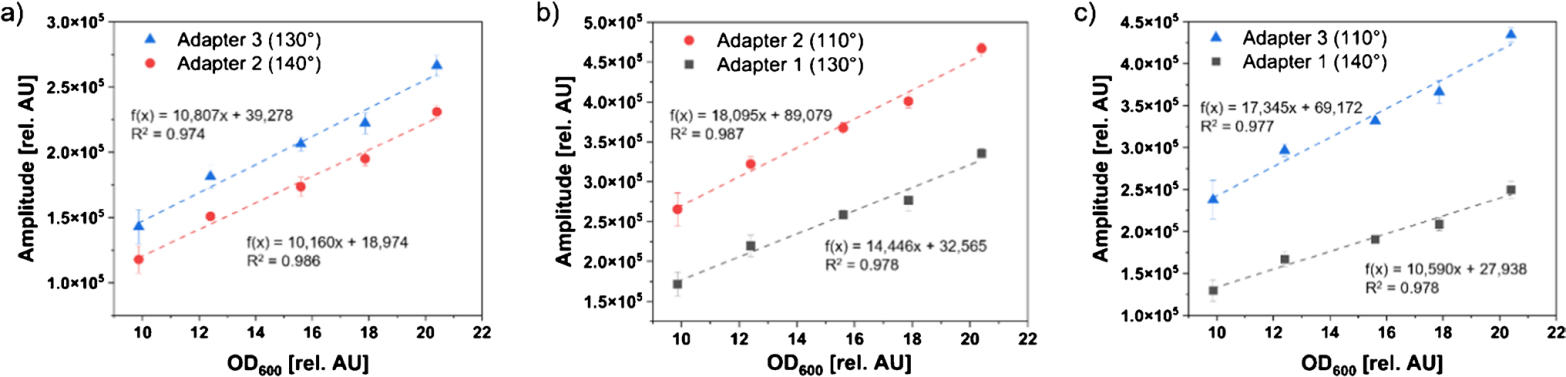
Influence of the measurement angle on the signal intensity. Mean signal intensities and standard deviations of three separate *E. coli* solutions measured with the SFR vario are shown. **a** Thick adapter with a measurement angle of 130° and a medium adapter with a measurement angle of 140°. Both measurements share the same waveguide length of 12.44 mm. **b** Medium adapter with a measurement angle of 110° and thin adapter with a measurement angle of 130°, sharing a waveguide length of 10.70 mm. **c** Thick adapter with a measurement angle of 110° and medium adapter with a measurement angle of 140°. Both measurements share the same waveguide length of 11.68 mm

In order to examine the influence of the measurement angle on the detected signal intensity, *E. coli* solutions in the OD_600_ range of 10–20 rel. AU were measured inside the three adapters. As indicated in Table 1, these adapters share the same waveguide length for different measurement angles (e.g., 130° of the thin adapter and 110° of the medium adapter), thereby allowing for the examination of the influence of the measurement angle on the signal intensity independently of the waveguide length. An increase in the measurement angle by 10° (Fig. 3a) resulted in a mean signal decrease of approximately 15.1% (± 2.1%) across the measurement range. Increasing the angle by 20° (Fig. 3b) and 30° (Fig. 3c) reduced the detected intensity by 31.2% (± 2.4%) and 43.4% (± 1.1%), respectively.

In addition to demonstrating elevated amplitudes across the entire OD_600_ range, the linear fits for smaller measurement angles exhibited a greater slope. With the measurement angle of 110°, a 38.9% higher slope was achieved in comparison to the angle of 140° (Fig. 3c). For biomass measurements, a higher slope in the calibration would be beneficial, since this would lead to an enhanced sensitivity [27]. Although online scattered light measurements with devices such as the SFR vario are typically conducted at 180° [15, 17] or 135° [4], the findings of this study suggest that the optimal measurement angle for the present application is 110°. Previous studies have shown that the horizontal measurement of scattered light at an angle of excitation and detection of 130° can improve the sensitivity while decreasing the SNR [19]. These findings indicate that modifying the measurement angle to 110° may result in enhanced sensitivity and SNR.

### Measurements of different microorganisms

To verify the ideal angle of excitation and detection for light scattering measurements inside the optical adapter, suspensions of model cell lines (*Escherichia coli*, *Bacillus subtilis*, and *Saccharomyces cerevisiae*) were measured across a broad OD_600_ range in the optical adapter. For this, cultures with the respective cell lines were harvested in stationary phase, washed, and then resuspended in PBS. Serial dilutions were prepared, and the signal intensities were measured using the SFR vario and the optical adapter 1 for all six angles.

**Fig. 4:**
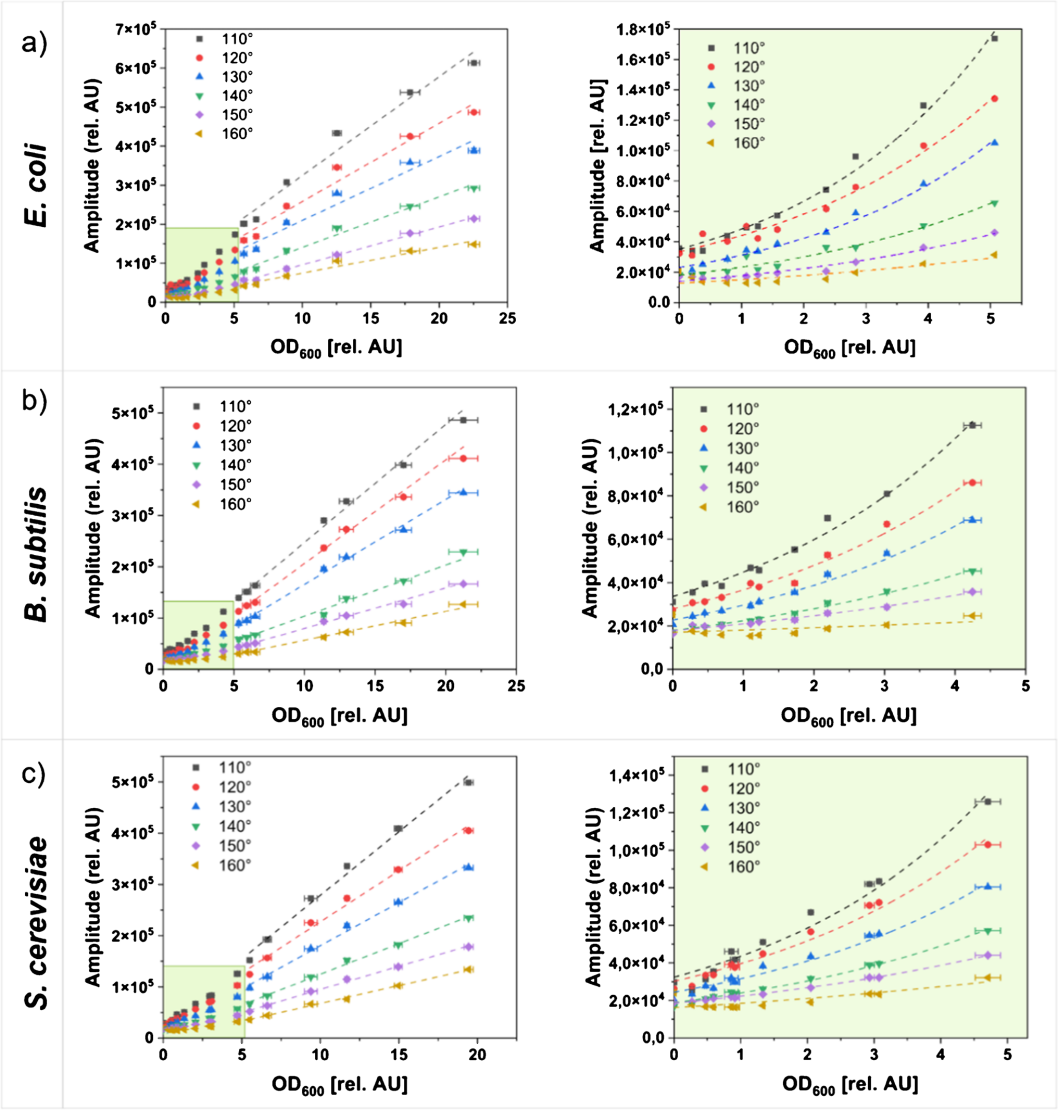
Scattered light signals obtained from cell suspensions in the stationary phase using the 3D-printed optical adapter. Detected signals of *E. coli* (**a**), *B. subtilis* (**b**), and *S. cerevisiae* (**c**) suspensions. For signals measured with an OD_600_ > 5 rel. AU., an exponential fit was applied. Linear fits were used for signals obtained at OD_600_ > 5 rel. AU

For all organisms tested, the angle of 110° exhibited the highest signal intensities as well as the highest slope of the respective linear fit. As the measurement angles increased, the intensity and slope of the respective fit decreased accordingly. For the microorganisms *E. coli* (Fig. 4a), *B. subtilis* (Fig. 4b), and *S. cerevisiae* (Fig. 4c), the obtained signals can be divided into two parts: In regions of lower OD_600_, no linear relation between the OD_600_ and the resulting signal was observed, which is why these areas are described by an exponential fit with the formula $y=a \bullet {e}^{b}$. For solutions with an OD_600_ > 5 rel. AU, however, linear fits were applied. Notably, in a recently published previous study, a similar behavior was observed [20].

Relevant literature describes that measurements of backward scattered light (measurement angles of 160–180°) exhibit enhanced linearity at higher cell concentrations, while measurement angles of 90° bring the advantage of higher sensitivity at lower cell concentrations [28]. For all microorganisms tested, higher signal intensities were observed in low OD_600_ ranges for the measurement angle of 110°, indicating that these areas are better measured by smaller measurement angles. Similarly, at higher OD_600_ values, the measurement angle of 110° demonstrated the highest signal intensities, while the linear fits also exhibited the highest slopes. In the broad OD_600_ range examined in this study, the linearity did not suffer—a result which is supported by the respective values of the regression coefficient of determination. As a further measure for linearity, the relative standard deviation of the slope (RSD_b_) was calculated for the OD_600_ range > 5 rel. AU, as described in the literature [29]. A detailed list of the coefficients of determination, the slopes, and the RSD_b_ values can be found in the supplementary information (Table SI3). For solutions of *B. subtilis* and *S. cerevisiae* measured with an angle of 110°, RSD_b_ values below 5% were calculated. For the measurements of *E. coli* solutions, no substantial differences were observed in the RSD_b_ values between the different measurement angles.

For the measurement of *E. coli* suspensions, a mean deviation of 36% (± 4.6%) was observed between the angles of 110 and 130° across the OD_600_ range. This finding aligns closely with the results reported in the preceding sections, as the disparity in waveguide length between the two angles, in conjunction with the measurement angle itself, resulted in variations of 6.9% and 31.2%, respectively.

In our previous work, a custom 3D-printed cultivation vessel was also described which can be mounted onto the SFR vario and be used to monitor the growth of microorganisms, leading to reductions in the noise-to-signal ratio of more than 80% in comparison to conventional vertical measurements in shake flasks [20]. We note that the waveguide pair of the excitation and detection angle of 110° of the optical adapter presented in this study could easily be transferred to the existing design, in order to further increase the sensitivity of the system.

## Conclusion

In this study, we present a 3D-printed optical adapter that can be used to assess the optimal measurement angle of light scattering measurements with a redirected light path. This adapter allows for measurements to be performed in a lateral orientation, instead of vertically through the vessel’s bottom. This optical adapter is compatible with the SFR vario device and features six measurement angles of excitation and detection—ranging from 110 to 160°—which facilitate a quick measurement of sample solutions with all angles.

This optical adapter was utilized to conduct scattered light measurements on various microorganisms, and the obtained signal intensities were matched to offline-determined OD_600_ values. Using solutions of the model organism *E. coli* and optical adapters with varying base thicknesses, we demonstrated that the waveguide length, as well as the measurement angle, both influence the obtained signal intensities. Furthermore, both of these factors were observed to affect the slope of the linear fits applied to the generated data, suggesting that shorter waveguides and smaller measurement angles lead to higher sensitivities. This was confirmed for selected microorganisms. The obtained data revealed that a measurement angle of 110° was optimal for the present application, and moving forward, that information can now be integrated into similar published designs for custom 3D-printed cultivation vessels in order to allow for more sensitive horizontal measurements of scattered light during microbial cultivation. Due to the potential to easily adapt designs for 3D-printed devices, the presented adapter can also potentially be modified to facilitate the assessment of measurement angles for cultivation vessels of varying sizes and geometries.

## Supplementary Information

Below is the link to the electronic supplementary material.

Supplementary Material 1 (DOCX 58.4 KB)

## Data Availability

All data is available from the corresponding author upon reasonable request.
