# Supplementary material for: Optimization of the angle for scattered light measurements in 3D-printed cultivation vessels
Source: Anal Bioanal Chem. 2025 Oct 2;417(25):5627–35. doi: 10.1007/s00216-025-06131-4 (PMC12528326; doi:10.1007/s00216-025-06131-4)
Supplement: Supplementary file 1 — Supplementary Material 1 (DOCX 58.4 KB) [file 216_2025_6131_MOESM1_ESM.docx]

# Supplementary Information

# Optimization of the angle for scattered light measurements in 3D-printed cultivation vessels

Nicolas Debener^1┼^, Louis Maximilian Kuhnke^1┼^, Sascha Beutel^1╪^ and Janina Bahnemann^2,3*╪^

^1^Institute of Technical Chemistry, Leibniz University Hannover, Hannover, Germany

^2^Institute of Physics, University of Augsburg, Augsburg, Germany

^3^Centre for Advanced Analytics and Predictive Sciences (CAAPS), University of Augsburg, Germany

*Correspondence:

janina.bahnemann@uni-a.de

^┼^ These authors contributed equally to this work and share first authorship.

^╪^ These authors contributed equally to this work and share last authorship.

ORCID

Sascha Beutel: <https://orcid.org/0000-0002-0983-9748>

Janina Bahnemann: <https://orcid.org/0000-0002-7008-1673>

**
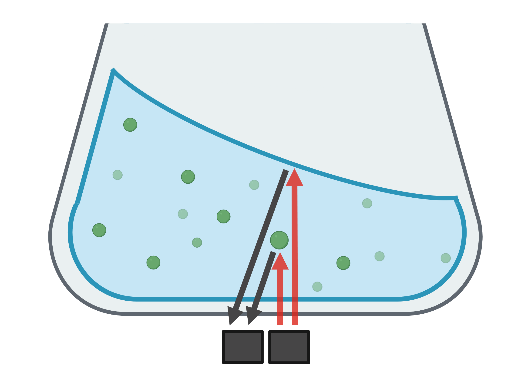
**

**Fig. SI1** Schematic illustration of reflections at the liquid-air interface within shake flasks (created using biorender.com)


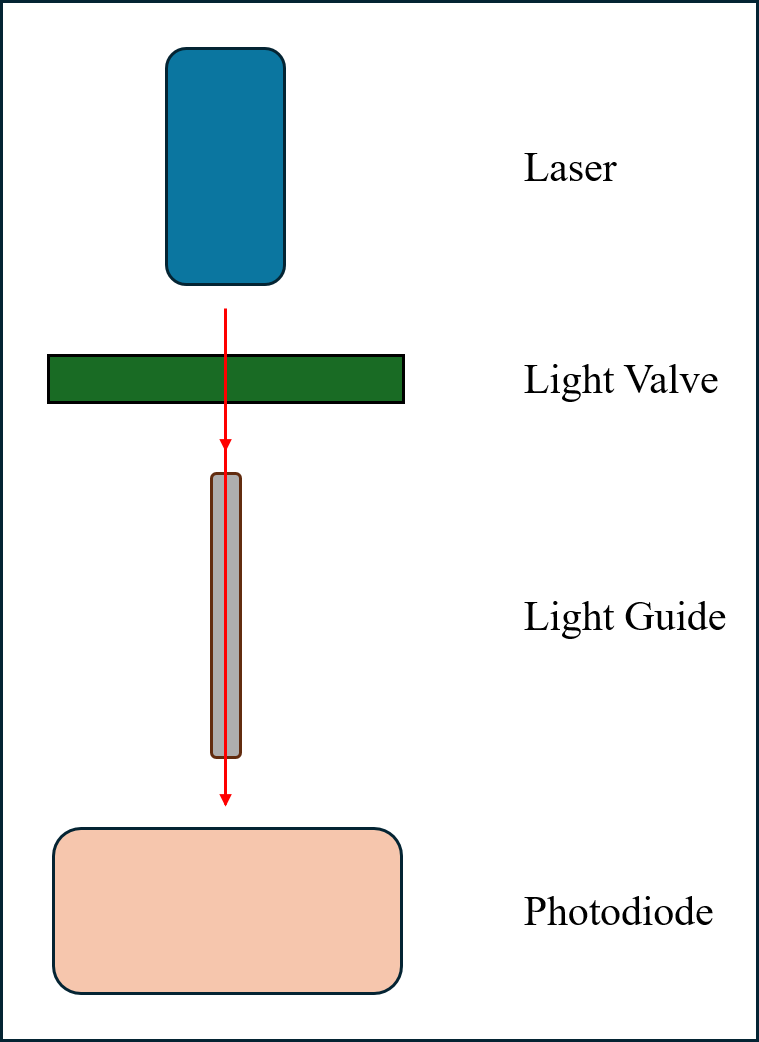


**Fig. SI2** Schematic illustration of the optical bench setup

**Table SI3** Slopes, coefficients of determination (R^2^) and RSD_b_ values of the linear fits applied to signal intensity values obtained from the measurement of solutions of different microorganisms

| *E. coli* | | | |
| --- | --- | --- | --- |
| Measurement angle [°] | Slope | R^2^ | RSD_b_ [%] |
| 110 | 25,303 | 0.972 | 8.469 |
| 120 | 20,039 | 0.971 | 8.695 |
| 130 | 16,386 | 0.959 | 10.381 |
| 140 | 12,950 | 0.983 | 6.528 |
| 150 | 9,707 | 0.996 | 3.210 |
| 160 | 6,606 | 0.962 | 9.899 |
|  |  |  |  |
| *B. subtilis* | | | |
| Measurement angle [°] | Slope | R^2^ | RSD_b_ [%] |
| 110 | 22,093 | 0.995 | 3.685 |
| 120 | 19,057 | 0.996 | 6.573 |
| 130 | 16,129 | 0.997 | 2.905 |
| 140 | 10,590 | 0.991 | 4.901 |
| 150 | 7,634 | 0.996 | 3.354 |
| 160 | 5,845 | 0.989 | 5.187 |
|  |  |  |  |
| *S. cerevisiae* | | | |
| Measurement angle [°] | Slope | R^2^ | RSD_b_ [%] |
| 110 | 24,531 | 0.991 | 4.700 |
| 120 | 19,934 | 0.991 | 4.859 |
| 130 | 16,831 | 0.994 | 3.816 |
| 140 | 11,945 | 0.996 | 3.225 |
| 150 | 9,014 | 0.997 | 2.810 |
| 160 | 6,964 | 0.997 | 2.844 |
|  |  |  |  |
